# Dexmedetomidine Attenuates Hypoxemia During Palliative Reconstruction of the Right Ventricular Outflow Tract in Pediatric Patients

**DOI:** 10.1097/MD.0000000000000069

**Published:** 2014-09-12

**Authors:** Qiang Chen, Wei Wu, Gui-Can Zhang, Hua Cao, Liang-Wan Chen, Yun-Nan Hu, Yan-Dan Chen

**Affiliations:** Department of Cardiovascular Surgery (QC, G-CZ, HC, L-WC, Y-NH, Y-DC); and Department of Anesthesia, Union Hospital, Fujian Medical University, Fuzhou, Fujian, P.R. China (WW).

## Abstract

The objective of this study was to investigate whether the α agonist dexmedetomidine has the ability to attenuate hypoxemia in pediatric patients undergoing palliative pulmonary artery reconstruction.

From January 2009 to January 2013, a total of 25 pediatric patients with Tetralogy of Fallot, pulmonary atresia (ventricular septal defect), or persistent truncus arteriosus (I) were enrolled in our study. Due to hypoplastic pulmonary arteries, all patients received palliative pulmonary artery reconstruction. During the perioperative period, they were allocated to receive either dexmedetomidine (bolus dose of 0.3 μg/kg followed by an infusion of 0.2–0.3 μg/kg/h, n = 15) or control drug (n = 10) intravenously. Any desaturation was recorded. Heart rate, mean arterial pressure, pulse oximetry, and arterial blood gas parameters were measured during the perioperative period.

There were no significant differences between the groups in hemodynamic variables. The arterial oxygen saturation and arterial blood gas parameters increased in the dexmedetomidine groups (*P* < 0.05).

These findings suggest that the injection of dexmedetomidine can attenuate hypoxemia during palliative pulmonary artery reconstruction in pediatric patients.

## INTRODUCTION

Cyanotic congenital heart defects (CHDs) such as Tetralogy of Fallot, pulmonary atresia (ventricular septal defect), and persistent truncus arteriosus (I) are anomalies that occur with a hypoplastic pulmonary artery. An “ideal” surgical treatment for such CHD consists of the restoration of antegrade blood flow in the lung as well as the repair of the CHD, if possible. However, in some circumstances, palliative procedures serve as important components of multistage surgical treatment of these complex anomalies. They also prepare the pulmonary circulation and the left ventricle for the subsequent complete repair of the CHD. However, according to our observations, early postoperative hypoxemia, even lower than that in the preoperative status, is an important complication of such surgery. Dexmedetomidine, a potent α adrenoceptor agonist, that dose-dependently reduces arterial blood pressure and heart rate (HR), decreases the hemodynamic and plasma catecholamine response.^1–4^ It has been used in a variety of ways, including as a sedative agent, anesthetic adjuvant, premedicant, and anxiolytic. The effects of dexmedetomidine on pulmonary vasculature in children have not been extensively studied. Some reports show that the pulmonary artery pressure and pulmonary vascular resistance are decreased, followed by sustained levels of dexmedetomidine.^5–7^ It is important to prevent an increase in pulmonary vascular resistance and to improve the distal pulmonary circulation in palliative pulmonary artery reconstruction. In this study, we aimed to investigate dexmedetomidine’s effects on those cyanotic CHD patients who had just undergone palliative pulmonary artery reconstruction.

## MATERIALS AND METHODS

The present study was approved by the ethics committee of Fujian Medical University, China and adhered to the tenets of the Declaration of Helsinki. Additionally, written informed consent was obtained from the patients’ parents.

### Patients

Between January 2009 and January 2013, a total of 25 pediatric patients with Tetralogy of Fallot, pulmonary atresia (ventricular septal defect), or persistent truncus arteriosus (I) were enrolled in the study at our institution. All of them were assessed as having diminutive pulmonary arteries (severely hypoplastic pulmonary arteries with central and peripheral stenosis) and not suitable for an arterioportal-shunt because of a diminutive pulmonary vascular bed. Palliative reconstruction of the right ventricular outflow tract without closure of the ventricular septal defect (antegrade palliation) was performed in these patients. There were 11 female and 14 male children. The patients were aged from 6 months to 18 months (mean ± standard deviation, 11.8 ± 3.5 months). Their weights ranged from 8.5 to 12 kg (9.1 ± 1.1 kg). All patients showed cyanosis and tachypnea at presentation. The mean preoperative arterial oxygen saturation ratio was 60% to 75% (68.1% ± 2.5%). Transthoracic echocardiography was used to confirm the patients’ diagnosis and describe the central portion of the pulmonary arteries. The McGoon ratio (ratio of the summed diameter of the right and left pulmonary arteries to the diameter of the descending thoracic aorta) was measured. The average of the McGoon ratio was 1.05 (range, 0.7–1.3). Aortopulmonary collateral arteries were present in these patients. The patient demographics and the specific diagnoses are listed in Tables 1 and 2.

**TABLE 1:**
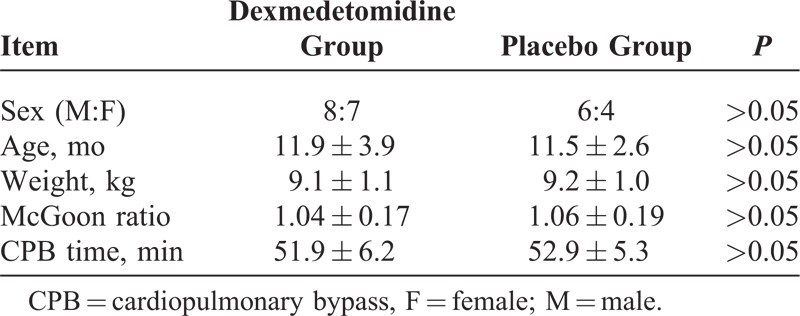
Clinical Data Collection From All Infants With CHDs in the Study

**TABLE 2:**
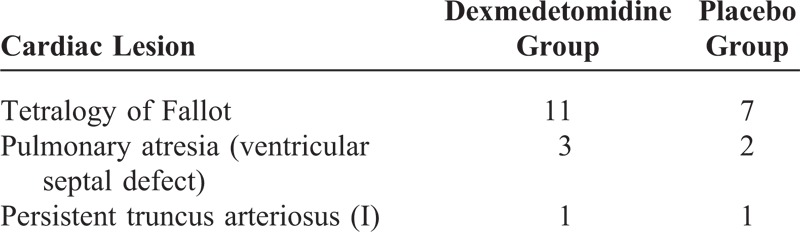
Details of Lesion and Operation

### Protocol

All operations were performed via a median sternotomy with cardiopulmonary bypass (CPB), mild hypothermia, and without cardiac arrest. A short longitudinal right ventricular incision opening the infundibulum was made. The ventriculotomy was extended across the main pulmonary artery trunk. After resection of infundibular muscle, a patch (autologous pericardial) as small as suitable was inserted to enlarge the right ventricular outflow tract. Additionally, in 2 children, a peripheral pulmonary artery stenosis was enlarged using an autologous pericardial patch. Three children had simultaneous surgical closure of a main aortopulmonary collateral artery.

All of the patients had similar general anesthetic techniques with oxygen, sevoflurane and intravenous propofol, fentanyl, and vecuronium. After operation, based on the different drug received by the patients, the patients were divided into 2 groups that contained a dexmedetomidine group (n = 15) and a control group (n = 10). No difference in age and body weight distribution was found in either group.

The dexmedetomidine group was started on an infusion of dexmedetomidine from 0.2 to 0.3 μg/kg/h with a loading dose 0.3 μg/kg intravenously. The dexmedetomidine was started intraoperatively following discontinuing CPB. The dosing was adjusted to maintain adequate sedation and analgesia during mechanical ventilation, for the weaning process. The dexmedetomidine was continued until discharge from extubation. Additional small boluses of propofol were available if needed for rescue sedation. Postoperatively, the control group was administered midazolam and fentanyl intravenously for sedation and analgesia.

In all patients, the following data were recorded: mean arterial pressure, HR, central venous pressure, pulse oximetry, and arterial blood gas parameters. The first record was obtained following discontinuing CPB (Time 1). After 10 minutes of starting dexmedetomidine or placebo, the second record was taken (Time 2). The third record was taken in the intensive care unit (ICU) while the continuous infusion was being administered (Time 3). The fourth record was drawn after extubation (Time 4). All the recorded clinical parameters are listed in Tables 3 and 4.

**TABLE 3:**
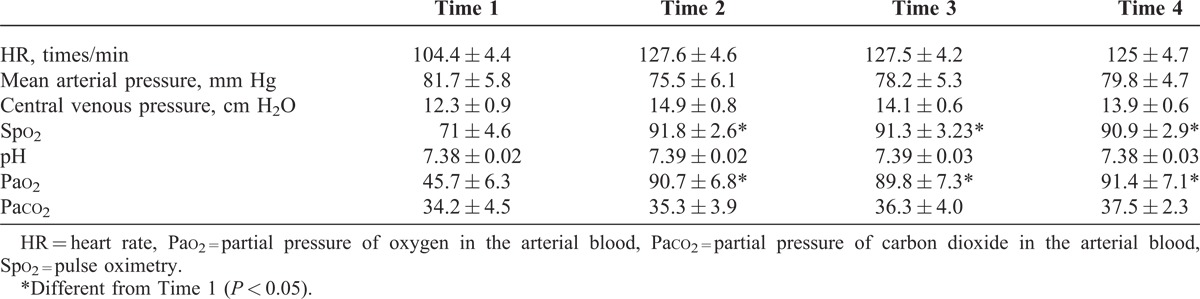
Clinical Parameters Before and After Palliative Pulmonary Artery Reconstruction Performed in the Infants With Cyanotic CHDs Associated With the Hypoplastic Pulmonary Artery in the Study (Dexmedetomidine Group)

**TABLE 4:**
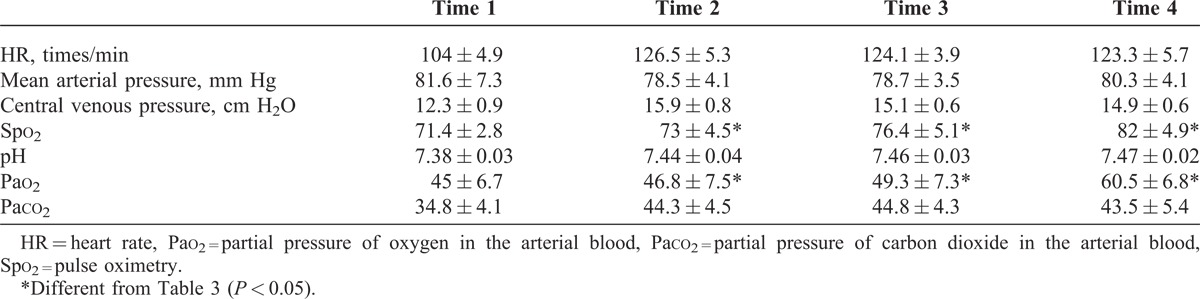
Clinical Parameters Before and After Palliative Pulmonary Artery Reconstruction Performed in Infants With Cyanotic CHDs Associated With the Hypoplastic Pulmonary Artery in the Study (Control Group)

### Statistical Analysis

Continuous data are presented as mean ± standard deviation and range. Clinical parameters between the 2 groups were compared with the independent sample *t* test. A *P* value of <0.05 was defined as statistical significance.

## RESULTS

From the information given in Table 3, it is clear that compared with the control group, the arterial oxygen saturation and arterial blood gas parameter Pao_2_ increased in the dexmedetomidine group (*P* < 0.05). The control group showed an elevated partial pressure of carbon dioxide in the arterial blood (Paco_2_ > 40 mm Hg), whereas the dexmedetomidine group did not have a clinically significant elevation of Paco_2_, although this was not statistically different. Hemodynamically, the dexmedetomidine group had periods of lower blood pressure than the control group but this was not clinically significant and did not require supportive therapy. No HRs <90 bpm were recorded in either group.

The time on mechanical ventilation was similar among groups, as was the length of stay in the ICU. Additional sedation and analgesia were required in 5 patients in the dexmedetomidine group.

The amount of dexmedetomidine required for sedation and analgesia ranged from 0.2 to 0.3 μg/kg/h. The duration of the dexmedetomidine infusion was 3 to 4 days. The dexmedetomidine was weaned off before discharge from extubation. No rebound hypertension and tachycardia were seen and the sedative and analgesic effects were not observed to be reversed acutely.

In the dexmedetomidine group, although the postoperative course was prolonged (median duration on ICU was 5 days) and complicated by congestive heart failure, clinically the 15 patients discharged improved substantially. The arterial oxygen saturation increased from 71 ± 4.6 to 90.9 ± 2.9 (*P* < 0.05) after the operation. The McGoon Index increased in the average from 1.05 to 1.65 and the arterial oxygen saturation was maintained between 81% and 92% during the follow-up.

## DISCUSSION

There are several therapeutic approaches to manage patients with Tetralogy of Fallot, pulmonary atresia (ventricular septal defect), or persistent truncus arteriosus (I). The severity of arterial hypoxaemia, intracardiac hemodynamics, and the development of a pulmonary vascular bed are the main factors that influence the choice of surgical strategy in these patients. The patients with diminutive pulmonary arteries remain a challenge. The hemodynamics of these lesions depends on a concomitant ventricular septal defect, the presence or absence of the volume of the pulmonary blood flow and the aorta pulmonary artery collateral circulation. In some circumstances, palliative procedures serve as an important component of multistage surgical treatment of these complex anomalies, which have been reported be Seipelt et al.^8^ Palliative pulmonary artery reconstruction in these pediatric patients results in an increase in pulmonary blood flow, offers substantially greater and more uniform growth of the pulmonary valve annulus and the central and peripheral portion of the pulmonary arterial tree, improves arterial oxygen saturation, and increases angiogenesis of distal microvessels.^9–11^ This procedure also prepares the pulmonary circulation and the left ventricle for the subsequent complete repair of CHDs. However, early postoperative hypoxemia, even lower than the preoperative status, is an important complication of such surgery.

Another disadvantage of antegrade palliation is that it may result in excessive pulmonary blood flow and congestive heart failure immediately after operation. An important limiting factor for postoperative excessive pulmonary blood flow is the pulmonary artery size itself. We managed to achieve an adequate pulmonary blood flow with the palliative right ventricular outflow tract reconstruction in several patients by making the diameter of the right ventricular outflow tract 70% of that of the normal pulmonary annulus.^12^ Our experience has found that postoperative hypoxemia is an important complication of this surgery. However, the reason for this is not understood. Several factors influence perioperative hypoxemia that was related to pulmonary dysfunction before operation, longer extracorporeal circulation time (≥2 hours), transfusion, hypoalbuminemia, and pulmonary infection. Ruling out the reason mentioned above, maybe the small reconstruction of the right ventricular outflow tract is one reason. Cardiac output and forward flow is dependent on a low pulmonary vascular resistance being maintained perioperatively. Surgical stimulation and early increased blood flow to the lungs may lead to increased pulmonary vascular resistance that may result in postoperative hypoxemia. Therefore, the routine sedation and analgesic medications used in those patients who received antegrade palliation were considered indispensable.

The use of dexmedetomidine has been approved for sedation in critically ill patients in multiple settings including the ICU, operating suite, cardiac catheterization laboratory, and during radiology. Dexmedetomidine also has been reported to reduce the stress response to surgery and intensive care.^13–17^ In mechanically ventilated ICU patients, dexmedetomidine has been shown to reduce the duration of delirium and coma while providing adequate sedation.^18^ One study demonstrated that dexmedetomidine was a useful agent for managing mechanical ventilation after cardiothoracic surgery and another study concluded that dexmedetomidine administered intraoperatively attenuated the hemodynamic and neuroendocrine responses to surgical trauma and CPB.^19,20^ Tokuhira et al^21^ reported a retrospective review on 14 pediatric patients who had undergone a Fontan procedure for congenital heart disease. The results of their study showed that dexmedetomidine can prevent an increase in pulmonary vascular resistance and indicates some potential advantages.^21^

In our study, the goals of the management of the antegrade palliation procedure patients were preventing the increase in pulmonary vascular resistance by maintaining adequate sedation and analgesia, preventing the production of excess endogenous catecholamines, and maintaining a calm anxiety-free patient.^22,23^ As shown in the previous reports, pulmonary artery pressure and pulmonary vascular resistance initially decreased, followed by a sustained plasma level of dexmedetomidine. Most patients after cardiothoracic surgery and after exposure to CPB have markedly increased circulating catecholamines, which may contribute to an initially elevated and labile pulmonary vascular resistance and pressure. Dexmedetomidine’s effect on the vasculature may have the potential to cause vasodilation through activation of central α_2_ adrenergic receptors that may result in pulmonary vasodilation.^24^ Ebert et al^5^ showed that pulmonary artery pressure and pulmonary vascular resistance initially decreased, followed by a sustained increase in plasma levels of dexmedetomidine to >1.9 ng/mL in a biphasic response to increasing doses of dexmedetomidine in adult volunteers. Although, in these patients, there were no accurate pulmonary vascular resistance measurements made with/without a dexmedetomidine infusion, we can comment on the effect of the elevated arterial oxygen saturation and arterial blood gas parameter Pao_2_ by the infusion of dexmedetomidine. The goal was to alleviate the pulmonary vascular resistance and provide adequate sedation and hemodynamic stability without respiratory depression, which we believe was achieved.

Elevated Paco_2_ potentially could cause pulmonary vascular resistance to increase, lead to reduced pulmonary flow, and worsen hypoxemia. The dexmedetomidine group did not have any periods of respiratory depression, as demonstrated by a clinically significant elevated Paco_2_, therefore, demonstrating the hypothesis that this might be a better sedation technique for this group of patients, by preventing respiratory depression and the concomitant potential increase in pulmonary vascular resistance.

The major potential adverse effects of dexmedetomidine in postcardiac surgical patients are the result of its action of modulating the release of catecholamines and the reduction in the stress responses to the adverse effects of surgery. Many reports showed that dexmedetomidine patients developed a slowing of the HR, which were treated easily with drugs such as exogenous catecholamines and atropine of glycopyrrolate, or was resolved spontaneously when weaning from dexmedetomidine.^25–27^ Fortunately, we did not encounter such a phenomenon in our study. Jooste et al^28^ reported that rapid intravenous bolus administration of dexmedetomidine resulted in a transient increase in systemic and pulmonary pressure and a decrease in HR, although it was clinically well tolerated.^28^ Friesen et al^29^ compared the hemodynamic response with dexmedetomidine loading dose in children with and without pulmonary hypertension. They observed that the dexmedetomidine initial loading doses were not associated with pulmonary vasoconstriction and hypertension, even in children with pulmonary hypertension.^29^ So, we chose to use a low loading dose and infusion rate on the lower end of the dosing regimens used in clinical anesthesia practice (loading dose of 0.3 μg/kg and an infusion of 0.2–0.3 μg/kg/h). The bradycardia found in some patients in the dexmedetomidine group did not appear to be indicative of significant clinical symptoms. Hypovolemia from blood loss during the perioperative period is poorly compensated for and therefore must be treated aggressively. Maintenance of a good volume status and close monitoring of the HR is essential if dexmedetomidine is to be used in such post cardiac surgical patients.

Like any retrospective study, there is bias associated with data collection and incomplete data for some patients. As a result of these 25 successful cases, our experience is still limited, and longer follow-ups are needed in future research. This study was limited to one institution, and other institutions may find different results.

In conclusion, the results of this retrospective review of the role of dexmedetomidine in the management of the antegrade palliation surgical pediatric patients indicates some potential advantages over current sedation and analgesia techniques. Our results showed that dexmedetomidine might attenuate pulmonary artery reflexes and improve perioperative hypoxemia during palliative reconstruction of the right ventricular outflow tract in pediatric patients.
